# Supplementary material for: Investigation on Antioxidant Activity and Different Metabolites of Mulberry (Morus spp.) Leaves Depending on the Harvest Months by UPLC–Q-TOF-MS with Multivariate Tools
Source: Molecules. 2023 Feb 17;28(4):1947. doi: 10.3390/molecules28041947 (PMC9961089; doi:10.3390/molecules28041947)
Supplement: Supplementary file 1 [file molecules-28-01947-s001.zip › molecules-2177584-supplementary.pdf]

SUPPLEMENTARY MATERIALS

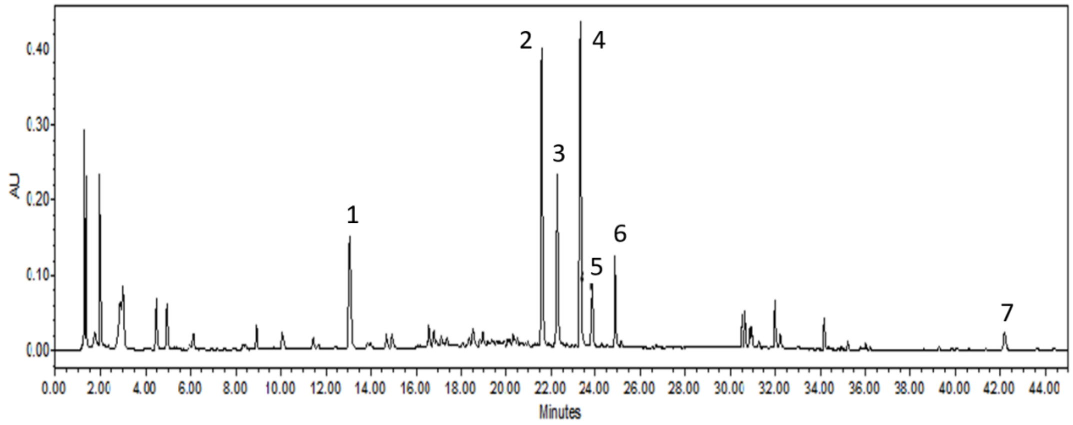

Figure S1. Typical UPLC chromatogram detected at 254 nm, and the main compounds 1 to 7 in the figure are chlorogenic acid, rutin, isoquercitrin, quercetin-O-acetyl glucoside, astragalin, kaempferol acetyl glucoside, and linolenic acid, respectively.

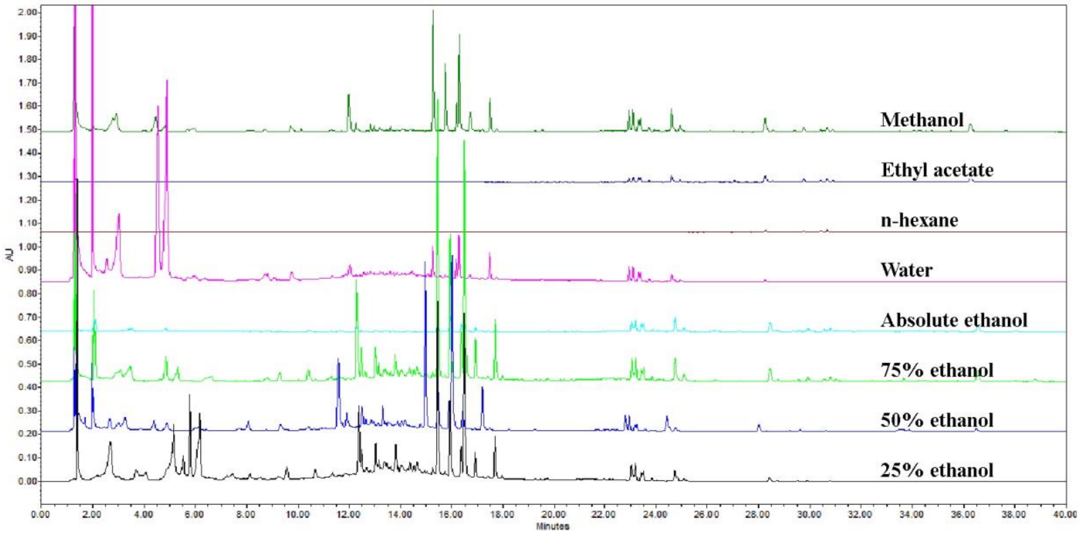

Figure S2. The UPLC results of the extracts of mulberry leaves with different solvents.

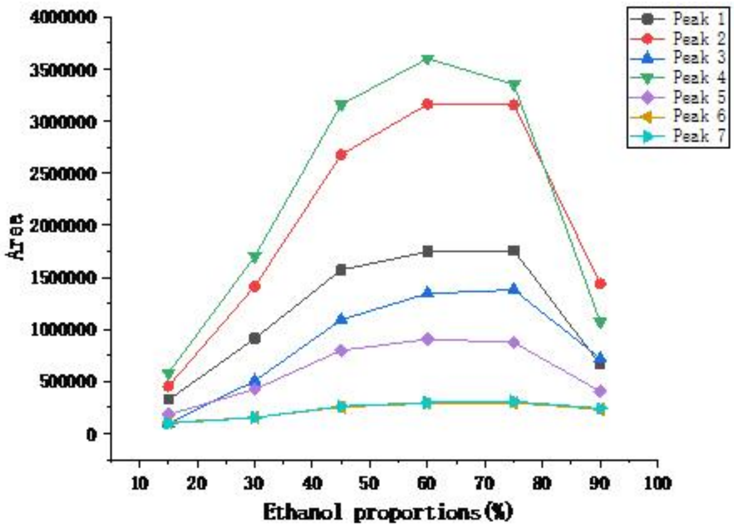

Figure S3. The effect of ethanol concentration on the extraction (peak area) of different compounds.

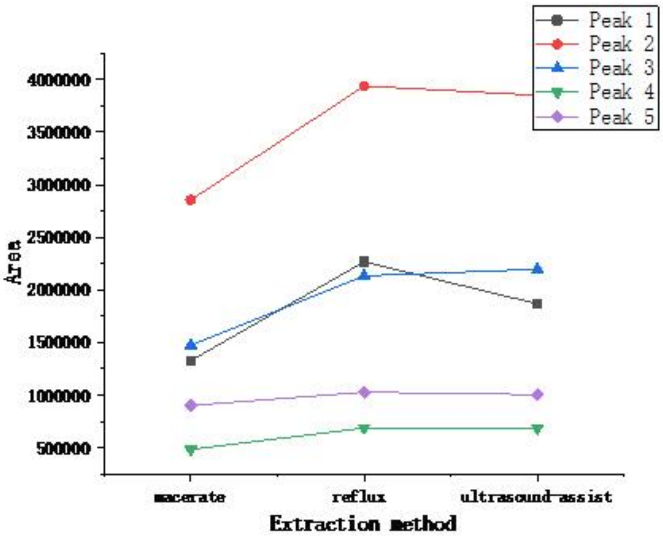

Figure S4. The effect of extraction method on the extraction (peak area) of different compounds.

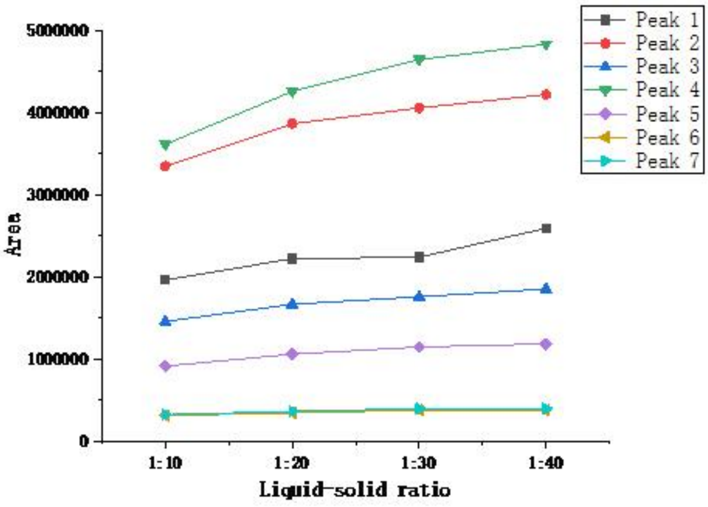

Figure S5. The effect of liquid-solid ratio on the extraction (peak area) of different compounds.

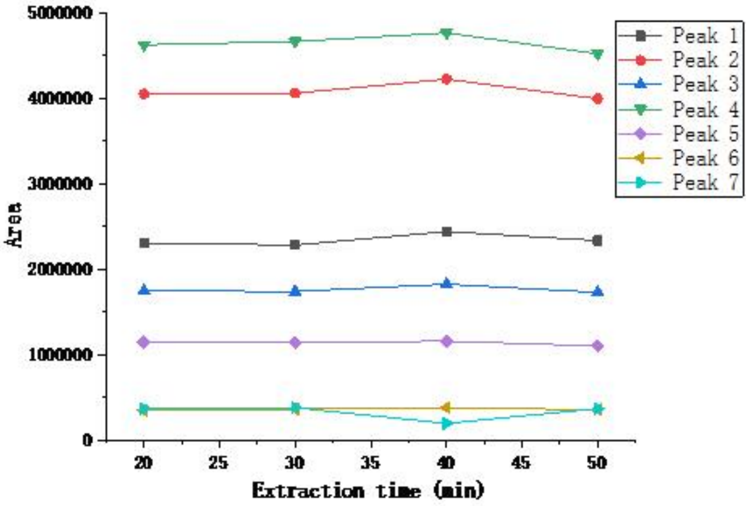

Figure S6. The effect of extraction time on the extraction (peak area) of different compounds.

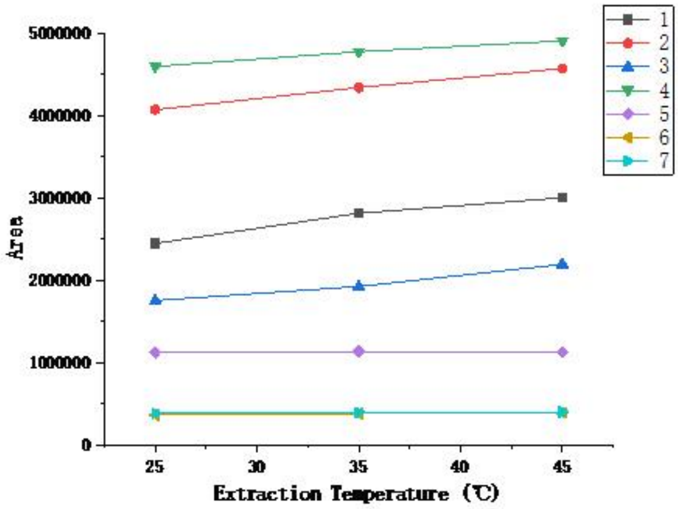

Figure S7. The effect of extraction temperature on the extraction (peak area) of different compounds.
